# The management of the faeces passed by under five children: an exploratory, cross-sectional research in an urban community in Southwest Nigeria

**DOI:** 10.1186/s12889-017-4078-1

**Published:** 2017-02-08

**Authors:** Olufemi Oludare Aluko, Olusegun Temitope Afolabi, Emmanuel Abiodun Olaoye, Adeyinka Daniel Adebayo, Seun Oladele Oyetola, Oluwaseun Olamide Abegunde

**Affiliations:** Department of Community Health, Obafemi Awolowo University, Ile-Ife, Nigeria

**Keywords:** Child faeces disposal;safe management of faeces, Faeces management chain, Childhood illnesses, Sanitation and hygiene, Nigeria

## Abstract

**Background:**

Safe management of faeces (SMoF) and environmental contamination by faecal pathogens have been extensively researched although the SMoF in under-five children has been perennially neglected perhaps due to the misconception that it is harmless. This research, therefore, studied the situation, to determine the magnitude and dimensions of the problem aimed at making policy level stakeholders aware of child faeces management systems and so, inform evidence-based implementation of child and health-related programmes in Nigeria.

**Methods:**

The study utilized an exploratory cross-sectional design and a multi-stage sampling technique to identify 300 respondents from 12 randomly selected streets from 4 wards in Ife central local government area. The study collected data with a pretested questionnaire which included direct observations of child defecation practices and existing toilet facilities. Cleaned data were analyzed by IBM-SPSS version 20 with child faeces management outcomes as the dependent variable.

**Results:**

The mean age of respondents’ and monthly income (mode) were 30.8 ± 7.5 years and ₦10,000.00 ($28.60). Most respondents were mothers to the under five children (84.7%), had a secondary education (72.0%) and were semi-skilled (57.0%). The caregivers had access to improved water sources (93.7%), improved toilets (64.3%), with 64% and 53.7% having above average scores in knowledge and attitudes, respectively. In the study, 19.7% and 69.0% of caregivers practiced safe disposal of faeces passed by the under five child during the day and at night respectively, though most caregivers (94.3%) omitted steps in the safe management of child faeces chain. The under five diarrhoea prevalence rate was 13.7% and unsanitary passage of child faeces is associated with four folds likelihood of having diarrhoea (*p* = 0.001). The caregivers whose under five children practiced safe sanitation were rich (*p* = 0.009) and knowledge was significantly associated with ownership of household toilet (*P* = 0.037), night faeces management chain practice (*P* < 0.001) and disposal of anal cleaning materials (*P* = 0.002). Handwashing was significantly associated with household toilet (*P* < 0.001), wealth (*P* < 0.001), under five child defecation preferences during the day (*P* < 0.001) and at night (*P* = 0.008).

**Conclusion:**

The high knowledge and positive attitudes exhibited by the caregivers were at variance with practice. Where under five children defecate during the day were influenced by the disposal of their anal cleaning materials, distance to the toilet and caregivers’ education. The findings highlight the dangers of unsanitary disposal of child faeces and the need to strengthen the related policies that can increase caregivers awareness and practice at all levels and in all livelihood domains.

**Electronic supplementary material:**

The online version of this article (doi:10.1186/s12889-017-4078-1) contains supplementary material, which is available to authorized users.

## Background

Environmental sanitation in practice applies the physical and biological principles to improve and control factors in the environment. Sanitation is an important foundation required towards the protection of public health and human welfare, which has assumed prominence in the international development agenda as a basic human right [1–3].

In 2013, the Nigeria Demographic and Health Survey (NDHS) revealed that households in Gombe state (Northeast Nigeria) has the highest access to improved sanitation facilities (66.5%) while those in Zamfara state (Northwest Nigeria) has the lowest access (6.9%) to improved sanitation facilities [4]. In addition, 30.1% households had improved, non-shared toilet facilities being used by 34.0% of the population with coverage skewed towards urban residence [4]. In Osun State, where this study occurred, only 16.1% of households had access to improved sanitation facilities, mostly in urban residential areas being used by 19.2% of the population [4]. As at 2015, the proportions of people that used improved (29%), shared (24%), other unimproved (22%) toilet facilities were below those required to meet the sanitation target of the Millenium Development Goal (MDG) seven, that sought to halve the proportion of people without sustainable access to basic sanitation in Nigeria [5]. The sanitation indices in Nigeria contributed to the poor human development index, typified by the high infant mortality rate (72.7 per 1000 live births) and under five mortality rate (157 per 1000 live births) [6]. The above statistics predisposes young children, who tend to defecate in areas where other susceptible children are exposed while learning to walk or playing in their premises, with frequent hands to mouth contacts, thus increasing the prevalence of faeces related diseases in such places and the risk of environmental enteropathy [7–9].

In 2012, the Wold Health Organisation/UNICEF Joint Monitoring Programme (JMP) reported that about 121.9 million Nigerians lacked access to improved excreta disposal facilities while 38.8 million Nigerians practice open defecation. This meant that only 28% of Nigerian households had access to improved sanitation conveniences. The analysis of the Nigeria’s National Demographic Health Survey (NDHS), 2013 data, authored by Water and Sanitation Program of the World Bank reported that only 23% of households dispose of their youngest child’s faeces in an improved sanitation facility [10]. The prevalence of risky disposal of children’s faeces in Nigeria is higher among households without access to improved toilet, probably among dwellers in rural areas and urban slums. These are where poorer households, and those that practice open defecation predominate [10]. Safe excreta disposal in children is equally as important as in adults [11, 12], though efforts have been concentrated on promoting the construction, ownership and use of adult toilet facilities globally. The safest way to dispose of faeces of an under five child is to help him/her use the toilet or latrine. However, for the very young child, the faeces could be rinsed into the toilet or buried, which is synonymous to cat sanitation in the first instance instead of open defecation [11–13]. Unsafe faeces disposal practices include those left in the open, thrown into the garbage, put/washed/rinsed into open drains, eaten by animals where faecal pathogens predominate and spread, from multiple environmental media to human populations [14].

The World Health Organisation (WHO) attributed 88% of diarrhoea associated mortality in the world, to unsafe water, poor sanitation, or hygiene. Over 99% of diarrhoea, associated mortality occurred in developing countries while about 8 in 10 deaths occur in children [15]. In Nigeria, the diarrhoea prevalence rate (18.8%) is one of the highest in Africa and above the Sub-Saharan Africa average of 16%. Diarrhoea accounted for over 16% of child deaths in Nigeria and an estimated 150,000 deaths mainly among children under five annually. This is caused predominantly by poor sanitation and poor hygiene practices, especially when the faeces of 47% of children Under five years old in Nigeria is disposed of through unsanitary methods [10]. In the context of this research, the caregiver was any adult person, who could be a family member or paid helper that cares for a child between 1 and 59 months old, for at least two consecutive months. Hence, caregivers could be grandparents, parents, aunts, formal and informal institutions such as a creche, where they are catered for. From available statistics, the practice of safe child faeces management remains low in Nigeria and factors associated with the practice of caregivers have only been documented in a few literature [10, 16]. The study, therefore, explored the magnitudes, dimensions and determinants of child faeces management in an urban city in Nigeria.

## Methods

### Description of the study location

The study was conducted in Osun State in South-western Nigeria. Osun State has a tropical climate with distinct wet and dry seasons. The study took place in Ile-Ife, the headquarter of Ife Central and Ife East Local Government Areas (LGAs). Ile-Ife is bounded by Ife North, Ife East, Ife South and Atakunmosa West LGAs.

### Study design

The study utilized an exploratory cross-sectional design.

### Sample size determination

The minimum sample was calculated by using the formula for estimating single proportions [17];

$$ \mathrm{n} = {\mathrm{Z}}^2\mathrm{P}\ \left(1\hbox{-} \mathrm{P}\right)/{\mathrm{d}}^2 $$

n =Z²P (1-P)/d². n = minimum sample size

Z = standard normal variate, at 1.96, which corresponds to the 95% confidence level.

P = 23%, the proportion of under five caregivers that practice improved excreta disposal in Nigeria [10].

d = degree of accuracy desired or maximum allowable margin of error, it was set at 5% (0.05).

This gave a sample size 273, which was increased to 300 with 10% added for attrition, inappropriately filled or missing questionnaires.

### Sampling technique

The study utilized a multi-staged sampling technique. Ife Central LGA has eleven wards from which four were selected by simple random sampling. This was followed by the selection of three streets from the respective sample frame by simple random sampling. Houses in the selected streets were identified by systematic sampling method. Trained enumerators were assigned clusters of households delimited by road networks and footpaths. In selected houses, if there were more than one eligible households, simple random sampling was used to identify the index household that participated in the study. Only one household is eligible to be included in the study per house.

Each enumerator started at the proximate house, closest to the main road, soliciting every third household in a clockwise manner until the minimum sample for the street has been covered, according to the works of Russel et al. [18]. Three hundred and fifty-nine households were approached out of which 7.5% (*n* = 27) were not eligible by the inclusion criteria while 8.9% (*n* = 32) declined to participate, though eligible. A total of 25 households were recruited per street.

### Inclusion criteria

For caregivers to be enrolled in the study, they should have satisfied the conceptual definition of caregivers provided for the study; be willing to participate and be resident or working within the randomly selected households in the study area.

### Data collection and analysis

The study took place between February and April 2016. The study utilized a pre-tested questionnaire (refer to Additional file 1), which contained sections on socio-demographic information, knowledge, attitudes and practices on child faeces passage, transport, disposal and hygiene of children and caregivers. A direct observation was used to document practices on the child faeces management chain (faeces passage, transportation, transport tools, disposal, hygiene of the transport tools and handwashing), where the under five children defecated during the period of data collection and observation. During the data collection period, if the index child in any of the enrolled households either defecate or defecating when the enumerators were there, then the process is recorded and reported in the study. The observations were not triggered but only happened and recorded during the data collection phase of the study. The study also observed and recorded the features of the household toilet facilities and presence of human excreta around the premises.

The diseases presented in this study were self-reported by the caregivers of under-five children, based on a 2-week recall period. However, the enumerators were trained to recognize the signs/symptoms and probed for each of the four diseases prior to making informed decisions. The completed questionnaires were checked and cleaned for completeness, accuracy and coded. The data were entered using EpiData 3.1 (EpiData Association, Odense, Denmark) and analyzed by using IBM-SPSS version 20. Socio-demographic characteristics and other univariate variables were presented by summary statistics using mean ± standard deviation and mode for continuous variables and frequency/percentages for categorical variables.

In this study, a household was defined as all persons who shared a cooking pot [19]. The questionnaire had 10 and 11 question items on knowledge and attitude variables. Prior to analysis, correct responses were coded as ‘1’ while incorrect responses were coded ‘0’ for knowledge questions. The respondents’ attitude was measured by 5 point Likert scale: “Strongly agree”, “agree”, “undecided”, “disagree” and “strongly disagree” and scored 5, 4, 3, 2 and 1, respectively. For knowledge and attitude variables, the aggregate scores were dichotomized into good and poor for knowledge, positive and negative for attitude scales, respectively. In this regard, the median (inter-quartile range) for attained knowledge score were 7 (6–8). So, a score below 7 was recoded as low and a score equal to or greater than 7 was considered high [20, 21]. Similarly, a score of 42 (39–45) was used to stratify the sum of attitude scores on child faeces management into positive and negative categories, respectively. In constructing the wealth categories, variables such as ownership of the house, and household items such as fridge, Television/video, paid satellite television, care/motorcycle, laptop, separate room for kitchen contributed towards the measuring scale. The presence of the household items was rated ‘1’ while the lack of these was rated ‘0’. To stratify into categories, 1–39%, 40–69% and ≥ 70% were rated poor, average and rich, respectively. For bivariate analysis, chi-square (Pearson) statistics was used to assess the type of relationship among pairs of under five child defecation practice indices, except otherwise stated, with the level of significance at <5%.

### Multivariate analysis

Following the guidance provided by Cronin et al., variables with a *p*-value of <0.25 in univariate analysis were included in the multivariate analyses model [22]. Variables with a *p*-value of <0.05 after backward elimination were retained in the final model.

### Outcome measures

As suggested by Curtis et al., faeces disposal was recoded into a binary outcome, “safe” and “unsafe,” depending on whether the practice is prone to faecal contamination of the environment [23]. In addition, handwashing was also dichotomized into ‘correct’ and ‘incorrect’ practice outcome while faeces management chain (involving faeces passage, transport, disposal, washing of faecal transport materials and hand hygiene) was stratified into ‘safe’ and ‘unsafe’ practice. ‘1’ was assigned where all the attributes were safe while ‘0’ was assigned to situations where any one step in the faeces management chain was not safe.

## Results

### Socio-demographic and economic characteristics of respondents

In the study, the average age (mean ± Standard deviation) and monthly income (mode) were 30.8 ± 7.5 years and ₦10,000.00 ($28.60). Most caregivers were females (276, 92%), and of Yoruba ethnicity (240, 80%). Also, most caregivers were mothers of the under five children (254, 84.7%), had at least secondary education (216, 72.0%) and were semi-skilled (171, 57.0%) (Table 1). In addition, the study showed that Moreover, two-fifth of respondents (124, 41.3%) were rich while others belonged to the medium (74, 24.7%) and poor (102, 34.0%) economic clusters, respectively (Table 2).

**Table 1:** Socio-demographic characteristics of caregivers of under five children

Description of variables	Categories	Frequency	Percent
Age; years (*n* = 300)	≤29	156	52.0
	≥ 30	144	48.0
Gender (*n* = 300)	Male	24	8.0
	Female	276	92.0
Marital Status (*n* = 297)	Single	17	5.7
	Married	265	88.3
	Divorced/separated/widow/er	15	5.0
Type of marriage (*n* = 292)	Polygamous	61	20.3
	Monogamous	215	71.7
	Single parenthood	16	5.3
Religion (*n* = 300)	Christianity	205	68.3
	Islam	84	28.0
	Traditional	7	2.3
	Others	4	1.3
Ethnicity (*n* = 296)	Yoruba	240	80.0
	Igbo	44	14.7
	Hausa	8	2.7
	Others	4	1.3
No of under five children caring for (*n* = 300)	1 under 5 child	184	61.3
	≥ 2 under 5 children	116	38.7
Relationship to under five child (*n* =280)	Mother	254	84.7
	Others	26	8.7
No of household members (*n* = 293)	≤ 4	151	50.3
	≥ 5	142	47.3
Number of bedrooms occupied (286)	≤ 3	261	87.0
	≥ 4	25	8.3
Respondents’higest educational attainment (*n* = 300)	No Formal Education/Primary	71	23.7
	Secondary	116	38.7
	Tertiary	100	33.3
	Others (Quranic/vocational)	13	4.3
Highest education of respondents’ spouse (*n* = 300)	No Formal Education/Primary	61	20.3
	Secondary	89	29.7
	Tertiary	115	38.3
	Others (Quaranic/vocational)	35	11.7
Occupation of respondents (*n* = 300)	Business/Commerce	43	14.3
	Civil Service	48	16.0
	semi skilled	171	57.0
	Others	38	12.7

**Table 2:** Economic indices of caregivers of under five children in the study area

Description of variables	Yes		No	
	Frequency	Percent	Frequency	Percent
Household owns residential building	92	30.7	208	69.3
House built with blocks	202	67.3	98	32.7
House floor cemented and/or tiled	191	63.7	109	36.3
Household has a functional fridge/freezer	155	51.7	145	48.3
Household has a functional TV/video sets	219	73.0	81	27.0
Household subscribed to paid satellite TV receivers	136	45.3	164	54.7
Household owns a car or motorcycle	169	56.3	131	43.7
Household has a generator/desktop or laptop computer	153	51.0	147	49.0
Household has separate room for kitchen	207	69.0	93	31.0
Household uses gas/electric cooker/kerosine as the main cooking energy source	208	69.3	92	30.7
Household shares bathroom/toilet with other households	141	47.0	159	53.0

### Knowledge of caregivers on child defecation and hygiene indicators

As shown in Table 3, 82% (247) of respondents knew about the sanitary handling of faeces of under five children while only 32.3% (97) had knowledge that faeces of under five children can not be used on farms as manure without adequate treatment. Moreover, 36% (109) respondents knew about correct handwashing process while about two-third respondents (126, 42%) knew about critical times for handwashing. The composite knowledge analysis, however, showed that six out of ten respondents (192, 64%) had good knowledge while 108 (36%) had poor knowledge on assessed sanitation and hygiene indices.

**Table 3:** Knowledge of caregivers on selected child defecation and hygiene variables on child defecation management practices

Description of variables	Correct response		Incorrect response	
	Frequency	Percent	Frequency	Percent
Sanitary ways of handling the faeces of under five children (*n* = 300)	247	82.3	53	17.7
Faeces of under five children could be used immediately as manure on farms (*n* = 300)	203	67.7	97	32.3
Child training to safety defecate (*n* = 300)	201	67.0	99	33.0
Correct process in hand washing (*n* = 300)	109	36.3	191	63.7
Critical times when hands should be washed (*n* = 300)	126	42.0	174	58.0
Times when under five children faeces should be safely disposed (*n* = 300)	266	88.7	34	11.3
Location for safekeeping of child’s potty (*n* = 300)	189	63.0	111	37.0
Diseases associated with poor management of under five faeces (*n* = 300)	261	87.0	39	13.0
With what should child potty be washed after use (*n* = 300)	216	72.0	84	28.0
The faeces of under five children is harmless (*n* = 300)	191	63.7	109	36.3

### Attitude of respondents on child defecation and hygiene practices

The findings of the study showed that 262 (87.3%) respondents believed that the use of improved toilet is mandatory for safe management of faeces (SmoF) of under five children and only 38 (12.7%) believed that the faeces of under five children is harmless when compared with those of adults. In addition, 283 (94.3%) respondents believed that under five children should be potty trained, right from infancy, while 80% (240) believed that disposal of child’s faeces on open dumps can cause faeco-oral diseases (Table 4). In the study, a little above average caregivers (161, 53.7%) had a positive attitude while 138 (46.3%) had a negative attitude on sanitation and hygiene variables related to the management of child defecation practices.

**Table 4:** Attitude of respondents on sanitation and hygiene variables on child defecation practices

Description of variables	Agree		Uncertain		Disagree	
	Frequency	Percent	Frequency	Percent	Frequency	Percent
Improved toilet is not mandatory since under five children can defecate in sanitary napkins and on soil	26	8.7	12	4.0	262	87.3
Safe disposal of child faeces and maintaining hygiene after cleaning is tiring	59	19.7	26	8.7	215	71.7
The faeces of an under five child is not harmful when compared with those of adults.	38	12.7	31	10.3	231	77.0
Hand washing after handling child’ faeces is not mandatory	16	5.3	09	3.0	275	91.7
Keeping the potty used for safe disposal of faeces by the under five children to water sources is essential to clean it thoroughly	55	18.3	18	6.0	227	75.7
It is not mandatory to wash hands of under five children with soap and water since child’s anus has been washed.	46	15.3	20	6.7	234	78.0
Faeces passed by under five child should be disposed of immediately after defecation	283	94.3	03	1.0	14	4.7
Since infancy, under five children should be potty trained	283	94.3	10	3.3	07	2.3
Safe disposal of faeces passed by under five children could be achieved even without ownership of improved household toilet	144	48.0	32	10.7	124	41.3
Buying potty is a waste of resources since the under five children can defecate around the premises	18	6.0	08	2.7	274	91.3
Disposing child’s faeces on open dumps can lead to faeco-oral diseases	240	80.0	33	11.0	27	9.0

### Child faeces management practices

#### Water supply and sanitation access by caregivers of under five children

In our study, most caregivers (281, 93.7%) had access to improved water sources, located outside their premises (177, 59.0%) with a modal travel time of five minutes for a return trip. Also, about two-thirds of caregivers (193, 64.3%) had access to improved toilet facilities while others used unimproved (70, 23.3%) toilet or practiced open defecation (36, 12.0%). In addition, the toilets of 154 (51.3%) caregivers were located outside their households premises and required a modal time of six minutes to use and/or disposed of the faeces passed by their under five children (Table 5).

**Table 5:** Description of water and sanitation facilities at households of respondents in the study area

Description of variables	Category	Frequency	Percent
Classification of household water sources by improvement status (*n* = 298)	Improved water source	281	93.7
	Unimproved water source	17	5.7
Distance of respondents’ household to safe water sources (*n* = 300)	within the household premises	123	41.0
	Outside the household premises	177	59.0
Average distance of water sources from households (*n* = 300)	5 min (mode)		
Sanitation facilities at household level by safety status (*n* = 299)	Improved toilet	193	64.3
	Unimproved toilet	70	23.3
	Open Defecation	36	12.0
Distance of respondents’ household to defecation facilities (*n* = 300)	within the household premises	146	48.7
	Outside the household premises	154	51.3
Average distance of households to toilet (*n* = 300)	6 min (mode)		
Usual under five child defecation practice during the day (*n* = 300)	Safe point defecation practice	59	19.7
	Unsafe point defecation practice	241	80.3
Usual under five child defecation practice at night (*n* = 300)	Safe point defecation practice	207	69.0
	Unsafe point defecation practice	93	31.0

### Excreta management chain behaviour among caregivers of under five children

#### Defecation practices of under five children in the study area

**Fig. 1:**
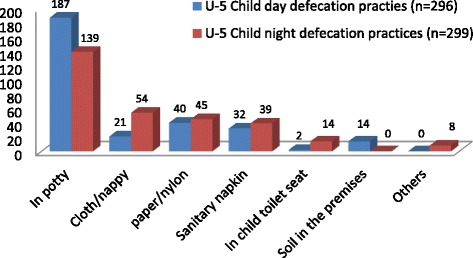
Day and night defecation practices of under five children in the study area

In the study, only 59 (19.7%) households practiced safe faeces disposal during the day in contrast to 207 (69.0%) that safely disposed of their children faeces at night (Table 5). In addition, the day and night preferences for under five children to pass faeces differs as shown in Fig. 1. The faeces passage arrangements in households of under five children showed that non -mobile (2, 0.7%) and crawling (16, 5.3%) children passed faeces in diapers while the use of potty commenced with the crawling under five children. In the same manner, the use of a toilet, and possibly toilet training, starts with children that can walk unaided (31, 10.3%) while most caregivers use the potty for containment of faeces passed by their wards (Table 6). The study further showed that the potty is mostly used by 187 (63.2%) caregivers during the day and 139 (36.5%) caregivers at night while sanitary napkin is preferred by 32 (10.8%) caregivers during the day and 39 (13.0%) caregivers at night. However, defecation by under five children was done on the premises only by one in twenty caregivers (14, 4.7%) during the day (Fig. 1).

**Table 6:** Faeces of under five children passage and disposal practices during the last defecation episodes in the study area

Variables description	Categories	Non-crawling (%)	Crawling (%)	Assisted walking (%)	Walking unaided (%)	Total (%)
Where were the faeces of under five children passed during the last defecation episode (*n* = 300)	Toilet	–	–	–	31 (10.3)	31
	Nappy	02 (0.7)	16 (5.3)	12 (4.0)	11 (3.7)	41
	Potty	–	03 (1.0)	23 (7.7)	152 (51.3)	178
	Nylon	–	–	01 (0.3)	05 (1.7)	06
	Paper	–	01 (0.3)	01 (0.3)	30 (10.0)	32
	Soil/ground premises	–	–	04 (1.3)	08 (2.6)	12
	Total	02 (0.7)	20 (6.6)	41 (13.6)	237 (79.6)	300
Where were the faeces of under five children disposed during the last defecation episode (*n*–288)	Rinse/emptied in the toilet	–	06 (2.0)	28 (9.3)	197 (65.7)	231
	Disposed with solid waste	01 (0.3)	12 (4.0)	08 (2.7)	24 (8.0)	45
	Buried in the premises	–	01 (0.3)	–	01 (0.3)	02
	Dilluted &spread in the premises	01 (0.3)	02 (0.7)	01 (0.3)	02 (0.7)	06
	Left in the open	–	–	01 (0.3)	03 (1.0)	04
	Total	02 (0.7)	41 (7.0)	38 (12.6)	227 (75.7)	288

### Transport of faeces passed by under five children in the study area

**Fig. 2:**
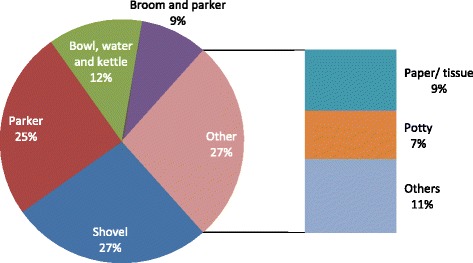
Faecal transportation processes for under five chidren

The potty was reported to be used for containment and transportation of the faeces of under five children by more than half of the respondents (154, 51.3%) while other transport devices included paper (16.7%) and nylon (5.3%) (Table 7). However, direct observation showed that caregivers used the shovel (27%), simple household packer (25%), bowl, broom and kettle (12%) among others (Fig. 2).

**Table 7:** Management of faeces passed by under five children by their caregivers

Description of variables	Category	Frequency	Percent
Transportation of child faeces (*n* = 279)	A potty	154	51.3
	Not applicable	59	19.7
	Paper	50	16.7
	Nylon	16	5.3
Routine disposal option of child faeces (*n* = 280)	Latrine/toilet	201	67.0
	In nearby bush/open dump	49	16.3
	In the household solid waste receptacle	23	7.7
	Open drainage	5	1.7
	Left in the open in the compound	2	.7
How child anus was cleaned after defecation (*n* = 299)	Rinsed with water	254	84.7
	Tissue paper/paper	21	7.0
	Paper	11	3.7
	Wipe with a clean section of soiled nappy/sanitary napkin	10	3.3
	Others	03	0.9
How under five child cleaning materials was disposed of after use (*n* = 297)	In toilet	163	54.3
	Cleaning water emptied in the environment	48	16.0
	Solid material disposed of in open dump	45	15.0
	Disposed with domestic solid waste	29	9.7
	No specific disposal method	12	4.0
Household own tool to manage child faeces (*n* = 300)	Yes	59	19.7
	No	241	80.3
Faeces management tools washed after use? (*n* = 241)	Yes	49	16.3
	No	11	3.7
Materials used in washing the faeces management tools (*n* = 49)	Soap and water	33	11.0
	Water only	16	5.3
Hand hygiene practices by caregivers after contact with under five child faeces (*n* = 299)	Wash hands with water and soap	241	80.3
	Wash hands with water only	43	14.3
	Clean hands with rag only	09	3.0
	Do not clean/wash hands after caring for the child	06	2.0

### Disposal of faeces of under five children after passage and transportation

Two-thirds of caregivers disposed of the faeces of their under five children in the toilet while the nearby bush/open dumps were used by 49 (16.3%) caregivers (Table 7). In addition, regarding the disposal of under five faeces, caregivers preferences varied with the mobility of under five children. The caregivers’ disposal of faeces by 197 (65.7%) under five children, especially those that walked unaided was done by rinsing and emptying into the toilet (Table 6). The study also showed that convenience (42%), health (28%) and cost (19%) influenced the existing defecation practices by the caregivers for their under five children. Besides, the anal cleansing of children was done most times by rinsing with water (254, 84.7%) and disposed of in the toilet by 54.3% caregivers (Table 7).

### Handling and safe keeping of faeces transportation and disposal tools

**Fig. 3:**
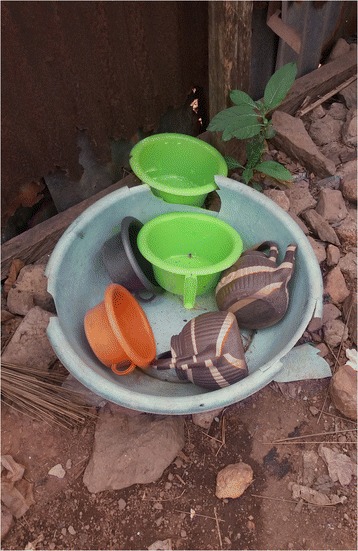
A receptacle for potties and anal cleansing water containers in the study area

In our study, only 59 caregivers, corresponding to 19.7% had dedicated tools for removal of children faeces from where passed, for disposal (Fig. 3). The tools were washed after use by only 49 (16.3%) caregivers with soap and water (33, 11%) and water only by 5.3% caregivers.

### Hand hygiene aftercare for under five children that passed faeces

Most caregivers (241, 80.3%) washed their hands with soap after caring for under five children that passed faeces, while other behaviour ranged from washing hands with water only (43, 14.3%) to non-cleaning/washing of hands afterward by 6 (2%) respondents. However, 94% of caregivers omitted at least one step in the process of ensuring SMoF.

### Period prevalence of selected childhood diseases

The 2-week period-prevalence of childhood diseases, among under five children showed that diarrhoea was prevalent at 41 (13.7%), followed by dysentery (7, 2.3%), cholera (5, 1.7%) and helminthic worm infestation (4, 1.3%).

### Relationship among child defecation management and associated variables

As shown in Tables 8 and 9, a significant association was found between handwashing practices and types of households toilets (*P* < 0.001), wealth (*P* < 0.001) defecation preferences of caregivers for under five children during the day (*P* < 0.001) and during the night (*P* = 0.008). In addition, knowledge was significantly associated with the types of households toilets (*P* = 0.037), faeces management chain (*P* = 0.032) and disposal of used anal cleaning materials (*P* = 0.002). In addition, diarrhoea was significantly associated with the day (*P* = 0.0001) and night (*P* = 0.0001) defecation practice by caregivers for under five children (Table 10). The odds of caregivers supported defecation of the under five children through improved toilet during the daytime, followed by handwashing were six times more than for those using the unimproved toilet, without handwashing. Also, most caregivers whose under five children practiced safe sanitation were rich (*p* = 0.009) (Table 8). In addition, the odds of defecation by under five children through improved toilet at night, followed by handwashing were three-folds than the odds of those using unimproved toilet/open defecation for disposal of under five children faeces without washing hands afterward. In like manner, the odds of faeces management by caregivers for under five children older than two years was five folds likelihood than for those who were older (Table 9). Moreover, the odds of under five children whose faeces were disposed of by unsanitary methods had four-fold likelihood of developing diarrhoea (Table 10).

**Table 8:** Relationship between socio-demographic by the management of under five children faeces variables in the study area

Variables	Categories	Sanitation chain by caregivers for under-5 children during the day		
Wealth categories		Unsafe	Safe	Total
	Poor	97 (95.1%)	5 (4.9%)	102 (34.0%)
	Average	72 (97.3%)	2 (2.7%)	74 (24.7%)
	Rich	114 (91.9%)	10 (8.1%)	124 (41.3%)
	Total	283 (94.3%)	17 (5.7%)	300 (100.0%)
	Chi square (χ) = 2.797; degree of freedom (df) = 2, *P*-value (*P*) = 0.247^a^			
Wealth categories		Sanitation chain by caregivers for under-5 children at night		
		Unsafe	Safe	Total
	Poor	49 (48.0%)	53 (52.0%)	102 (34.0%)
	Average	18 (24.3%)	56 75.7%)	74 (24.7%)
	Rich	26 (21.0%)	98 (79.0%)	124 (41.3%)
	Total	93 (31.0%)	207 (69.0%)	300 (100.0%)
	χ = 21.221; df = 2, *P* = 0.0001			
		Handwashing practices		
Variable	Category	Correct	Incorrect	Total
Types of household toilet	improved	87 (79.8%)	106 (56.1%)	193 (64.8%)
	Unimproved	22 (20.2%)	83 (43.9%)	105 (35.2%)
	Total	109 (36.6%)	189 (63.4%)	298 (100.0%)
	χ =17.062; df = 1; *P* < .0001; Odd ratio (OR) = 0.323; Confidence interval (CI) = 0.187-0.559			
Wealth	Poor	13 (12.7%)	89 (87.3%)	102 (34.1%)
	Average	19 (25.7%)	55 (74.3%)	74 (24.7%)
	Rich	77 (62.6%)	46 (37.4%)	123 (41.1%)
	Total	109 (36.5%)	190 (63.5%)	299 (100.0%)
	χ =64.764; df = 2; *P* < 0.0001			
Defecation practice of the under 5 child during the day	Improved	101 (40.6%)	148 (59.4%)	249 (84.4%)
	Unimproved	5 (10.9%)	41 (89.1%)	46 (15.6%)
	Total	106 (35.9%)	189 (64.1%)	295 (100.0%)
	χ ==14.870; df = 1; *P* < .0001^a^; OR = 5.596; CI = 2.138–14.648			
Defecation practice of the under 5 child during the night	Improved	100 (39.7%)	152 (60.3%)	252 (84.3%)
	Unimproved	9 (19.1%)	38 (80.9%)	47 (15.7%)
	Total	109 (36.5%)	190 (63.5%)	299 (100.0%)
	χ =7.210, df = 1, *P* = .008^a^; OR = 2.778; CI = 1.287–5.994			

^a^ = Fisher’s Exact Test

**Table 9:** Relationship between knowledge and selected variables on under five children defecation practices

Variables	Categories	Knowledge		
		Low	High	Total
Types of household toilet	Improved	78 (72.2%)	115 (60.2%)	193 (64.5%)
	Unimproved	30 (27.8%)	76 (39.8%)	106 (35.5%)
	Total	108 (36.1%)	191 (63.9%)	299 (100.0%)
	Chi square (χ) = 4.351; degree of freedom (df) = 1, *P* = 0.037; OR = 0.582; CI = 0.349–0.970			
Under-5 faeces management chain during the day the caregiver	Unsafe	98 (34.6%)	185 (65.4%)	283 (94.3%)
	Safe	10 (58.8%)	7 (41.2%)	17 (5.7%)
	Total	108 (36.0%)	192 (64.0%)	300 (100.0%)
	χ = 4.074; df = 1, *P* = 0.044; OR = 0.371; CI = 0.137–1.004			
Under-5 faeces management chain by caregiver at night	Unsafe	71 (40.3%)	105 (59.7%)	176 (58.7%)
	Safe	37 (29.8%)	87 (70.2%)	124 (41.3%)
	Total	108 (36.0%)	192 (64.0%)	300 (100.0%)
	χ = 3.483; df = 1, *P* = 0.062; OR = 1.590; CI = 0.975–02.592			
Disposal of the anal cleansing materials of under 5 children	Safe disposal	46 (28.2%)	117 (71.8%)	163 (54.9%)
	Unsafe disposal	61 (45.5%)	73 (54.5%)	134 (45.1%)
	Total	107 (36.0%)	190 (64.0%)	297 (100.0%)
	χ =9.552; df = 1, *P* = 0.002; OR = 0.471; CI = 0.291–0,762

**Table 10:** Relationship between daytime defecation practices and diarrhoea prevalence in the study area

Variables	Categories	Diarrhoea		
		No	Yes	Total
Defecation practice during the day	Unsafe	217 (90.4%)	23 (9.6%)	240 (80.3%)
	Safe	41 (69.5%)	18 (30.5%)	59 (19.7%)
	Total	258 (86.3%)	41 (13.7%)	299 (100.0%)
	Chi square (χ) = 7.525; degree of freedom (df) = 1, *P* = 0.0001; OR = 4.142 CI = 2.054–8.352.			
Defecation practice during the day	Unsafe	68 (73.1%)	25 (26.9%)	93 (31.0%)
	Safe	191 (92.3%)	16 (7.7%)	207 (69.0%)
	Total	259 (86.3%)	41 (13.7%)	300 (100.0%)
	Chi square (χ) = 19.949; df = 1, *P* = 0.0001; OR = 0. .228; CI = 115–0,452.			

The result of the multivariate analysis predicting under five child faecal passage preferences during the day (Table 11) and at night (Table 12) was done through binary logistic regression. The significant predictors of where under five children passed excreta irrespective of time of the day were the caregiver's occupation (civil servants), the age of index under five child, the transport and disposal of faeces whereas knowledge only predicts the passage of faeces preference by under five children at night (*P* < 0.05).

**Table 11:** Multivariate regression of variables predicting defecation passage preferences during the day for under five children by their caregivers

Factor	Odd ratio (OR)	95% confidence interval (CI)	*P*-value
Education			
None - Primary	1.920	0.793–4.646	0.148
Secondary and above	Ref		
Occupation			
Civil Servant	3.105	1.050–9.181	0.041
Other (semi-skilled/business)	Ref		
Age of index Under five children			
3–5 years	5.922	2.422–14.476	0.001
1–2 years	Ref		
No. of Under five children			
> 2	4.774	0.329–69.215	0.252
1–2	Ref		
Relationship of caregiver to the under-5 children			
Mother	1.568	0.545–4.509	0.404
Others (grandmother/paid carer)	Ref		
Location of the household toilet to the residence			
Outside the premises	1.954	0.819–4.660	0.131
Within the premises	Ref		
Knowledge			
Poor	2.326	0.993–5.446	0.052
Good	Ref		
Transport of faeces			
Unsafe	7.804	3.222–18.899	0.001
Safe	Ref		
Disposal of faeces			
Unsafe	2.593	1.126–5.969	0.025
Safe	Ref		
Handwashing process			
Incorrect	1.504	0.587–3.851	0.395
Correct	Ref

**Table 12:** Multivariate regression of variables predicting defecation passage preferences at night for under five children by their caregivers

Factor	Odd ratio (OR)	95% confidence interval (CI)	*P*-value
Education			
Secondary and above	1.580	0.784–3.185	0.201
None – Primary	Ref		
Occupation			
Civil Servant	2.896	1.059–7.919	0.038
Other (semi-skilled/business)	Ref		
Age of index Under five children			
1–2 years	1.385	0.763–2.517	0.284
3–5 years	Ref		
No. of Under five children			
> 2	1.970	0.401–9.680	0.404
1–2	Ref		
Relationship of caregiver to the under five children			
Mother	1.078	0.495–2.350	0.850
Others (grandmother/paid carer)	Ref		
Location of the household toilet to the residence			
Within the premises	1.630	0.842–3.154	0.147
Outside the premises	Ref		
Knowledge			
Good	3.495	1.802–6.780	0.001
Poor	Ref		
Transport of faeces			
Safe	2.250	1.092–4.640	0.028
Unsafe	Ref		
Disposal of faeces			
Safe	2.316	1.165–4.605	0.017
Unsafe	Ref		
Handwashing process			
Incorrect	1.298	0.649–2.595	0.461
Correct	Ref		

## Discussion

The safe management of child faeces require not just a sound knowledge and awareness but also the provisions of child-friendly facilities in houses where care is provided as the basic minimum. To manage faeces of under five children, caregivers are required to know about, and be able to apply appropriate preventive behaviour within the socio-cultural beliefs and precepts to promote public health. The study, therefore, explored faeces management practices of under five children in an ancient city in Nigeria, among caregivers who were ethnically and chronologically similar in socio-demographic characteristics but differentiated on their prevailing knowledge, attitudes, and practices.

### Knowledge of caregivers on defecation practices among under five children

Knowledge empowers when allowed to influence our attitudes and practices [24]. Many caregivers knew about the sanitary handling of faeces of under five children, correct handwashing practices and critical times when hand washing should be performed. The study identified two clusters of caregivers; those with low and high knowledge and attitudes. In our study, more than half caregivers had good knowledge and positive attitudes in contrast to the findings of Demberere et al., among mothers of under-five children in Mawabeni in Zimbabwe where they had poor knowledge and attitudes [25].

### Attitudes of caregivers on defecation practices among under five children

Positive attitude indicates the presence of the right dispositions on the behaviour that could aid sanitary management of child faeces. In our study, most caregivers thought the faeces of under five children was not as harmful as those of adults, in agreement with the findings of Bain and Luyendijk; Brown, Cairncross and Ensick and Gil et al. [14, 26, 27]. This contrasted with the situation in reality, where the faeces of under five children contains a higher number and diversity of pathogens [22]. In addition, only a little more than half of the respondents had a positive attitude and barring other confounders, will ensure that caregivers practice SMoF passed by the under five children, though the proportion that practiced SMoF of under five children could not confer herd immunity on other children in the study area. This is a concern for intervention since a positive attitude is a pre-requisite to safe behavioural change in the population, based on the explanations by Lanata et al. [8], that faeces of young children predominates in the environment where their peers are exposed and vulnerable to faeces related childhood illnesses. To avoid this situation. a positive attitude by caregivers during the formative years of the under five children are required to model their behaviour towards safe excreta management and hygiene practices.

### Access to water, sanitation and hygiene facilities in caregivers households

The importance of adequate and safe water to improved sanitation and hygiene practice has been discussed by authors [10, 28] and also in Burkina Faso by Curtis et al. [23] and in India by Sahay et al. [29], where access to water within the premises was associated with safe child faeces disposal. In this study, many caregivers had access to improved water supply, though located outside their premises which required about five minutes of travel time for a return trip, an additional burden on their numerous responsibilities. Therefore, limited availability of water within the premises of a few respondents, made it challenging to abide by best practices for child faeces management. Availability of safe water, within the households premises, enhances the caregivers’ ability to practice handwashing. Inadequate handwashing predisposes under five children to childhood diseases, probably through ‘make-shift’ arrangements, in agreement with the findings of Freeman et al. [30]. The above situation might be made worse by the lack of child-friendly facilities at the household level, to enable safe child defecation. Moreover, informal interaction with some caregivers revealed that the health promotion by nurses with pregnant women during antenatal visits excluded the information on SMoF for under five children, despite its associated risks.

In this study, about two-thirds of the caregivers had improved toilet facilities which should influence the health status of under five children., in agreement with Mosley and Chen [31] framework, which saw sanitation as a proximate determinant of child health and acknowledged its importance in providing a hygienic environment to the growing child.

### Under five faeces management practices by caregivers

According to World Bank/Unicef [10], pre-ambulatory under five children may not be able to use any toilet facility because of their age, stage of physical development and safety concerns irrespective of toilet access. This was in agreement with the findings in our study where about two-thirds of caregivers had access to improved toilet facilities whereas 19.7 and 69.0% among the under five children practiced safe defecation during the daytime and at night, respectively, in contrast to higher access to safe toilet facilities by the caregivers. The above findings were also in agreement with those of Demberere et al. [32] where 17% caregivers practiced safe disposal of children's faeces and with the findings of Miller-Petrie et al. [33] where ‘*child faeces are disposed of unsafely even among households with latrines*’ Besides, the six minutes of travel time to use and disposed of child’s faeces, outside the households premises contributed to the prevailing poor disposal practice.

There is a direct relationship between the age of the under five children and where they passed faeces, probably as the children age, they either learn about toilet discipline by the use of potty (Fig. 3) or disliked the sight of faeces. Moreover, the potty is used most of the time for the containment and transportation of children excreta, especially where the child has learned the habit of using the potty in defecation. Besides, the use of unsanitary transport media for faeces could predispose young children to enteric infection since they are mostly exposed to the ambient agents due to the time they spend roaming the environment and their habit of putting fingers and fomites in their mouths [34, 35]. Hence, caregivers should be knowledgeable about the dangers associated with allowing ‘*convenience*’ to determine the passage, transport, and disposal of faeces passed by the under five children.

In most cases, the use of tools dedicated to the management of under five child faeces was not considered important by caregivers in the study area. This should be improved to ensure that child faeces is being safely managed to improve their health and livelihood. Washing the tools after every use with soap and water was good, assisted by the presence of water, sanitation and hygiene facilities in the home environment.

In the study, hand hygiene was practiced by most caregivers with soap after caring for their ch children. This is good though the importance of sustainable hand washing at critical times by caregivers and for their under five children should be advocated for, more so when very few caregivers practiced SMoF. in the care of their under five children.

### Diarrhoea prevalence among under five children

Evidence from the works of Traore et al. and Miller-Petrie et al. [33, 35], confirmed that the presence of faeces, especially those of under five children in the premises has been found to be associated with diarrhoea and hospital admissions. Also, children are at a greater risk of diarrhoea if their caregivers disposed of their faeces in an insanitary manner according to Baltazar and Solon [36]. The above studies were in agreement with the findings in our study where under five children whose caregivers guided them on safe passage of faeces had a lesser risk of having diarrhoea. The presence of faeces in the premises of the under five children could be responsible for the high period prevalence of diarrhoea in the study, though the finding should be interpreted with caution as a result of seasonal variations in the prevalence of diarrhoea [4]. The study finding where handwashing was positively associated with ownership of improved toilet was also in agreement with that of Azage and Haile and Majorin et al. [37, 38], where handwashing was positively associated with household toilet ownership, wealth; day, and defecation practices of under five children at night while knowledge influences household toilet, composite faeces management chain and disposal of anal cleaning materials used to clean up under five children by their caregivers after defecation.

### Strengths and weaknesses of the study

The study is a population-based, exploratory and cross-sectional in design with the respondents randomly selected through multi-stage sampling technique in order to reflect their true characteristics. Hence, the findings can be generalised to the study population, especially in southwest Nigeria. One of the weaknesses of the study is that the data is partly dependent on self-reported information from caregivers of under five children. Though non-response persists in the data, conscious efforts were made to minimize it during data collection by engaging experienced enumerators, skilled in the art of data collection at the population level. However, causal effects can’t be adequately measured while disease prevalence was based on period prevalence, as at the time of the study, of course, which did not take into cognizance seasonal fluctuations which are time dependent.

## Conclusions

The good knowledge and positive attitude showed by caregivers were at variance with practice while the factors that predicted where under five children defecate during the day were, included where the child’ anal cleaning materials were disposed, the distance to the toilet facility to the residence and caregivers education. Hence, policy re-orientation, development of appropriate behavioural change communication guidelines should be developed into packages to address observed negative practices, mainstreamed and promoted through appropriate communication channels in Nigeria.

## Additional file

Additional file 1: Questionnaire on excreta management practices and diarrhoeal illnesses among under-five children in Ile Ife, Osun state, southwet Nigeria. The file contained the validated questionnaire used for data collection in the study area. (DOCX 55 kb)

## Abbreviations

JMP: Joint monitoring programme
LGAs: Local government areas
OD: Open defecation
SMoF: Safe management of faeces
