# Erratum to: The management of the faeces passed by under five children: an exploratory, crosssectional research in an urban community in Southwest Nigeria

**DOI:** 10.1186/s12889-017-4151-9

**Published:** 2017-03-10

**Authors:** Olufemi Oludare Aluko, Olusegun Temitope Afolabi, Emmanuel Abiodun Olaoye, Adeyinka Daniel Adebayo, Seun Oladele Oyetola, Oluwaseun Olamide Abegunde

**Affiliations:** Department of Community Health, Obafemi Awolowo University, Ile-Ife, Nigeria

## Erratum

After publication of the original article [1] it was brought to our attention that some of the authors’ final corrections were not incorporated in the online version of the paper.

1. In the ‘Background’ section, third paragraph, “Wold Health Organisation” has been corrected to “World Health Organisation”.

2. In Table 1, under column ‘Description of variables’, “Respondents’ higest educational attainment” has been corrected to “Respondents’ highest educational attainment”.

3. In Table 1, under column ‘Frequency’, a zero (0) has been added in front of single figures 7, 4, 8 and 4.

4. In Table 3, “Location for safekeeping of child’s potty” has been corrected to “Location for safe keeping of child’s potty”.

5. In Table 9, under column ‘Variables’, “Under-5” has been changed to “Under five”.

The above corrections have been incorporated in the original version of this article.
