# Supplementary material for: The management of the faeces passed by under five children: an exploratory, cross-sectional research in an urban community in Southwest Nigeria
Source: BMC Public Health. 2017 Feb 8;17:178. doi: 10.1186/s12889-017-4078-1 (PMC5299761; doi:10.1186/s12889-017-4078-1)
Supplement: Additional file 1: — Questionnaire on excreta management practices and diarrhoeal illnesses among under-five children in Ile Ife, Osun state, southwet Nigeria. The file contained the validated questionnaire used for data collection in the study area. (DOCX 55 kb) [file 12889_2017_4078_MOESM1_ESM.docx]

**DEPARTMENT OF COMMUNITY HEALTH**

**OBAFEMI AWOLOWO UNIVERSITY, ILE-IFE, OSUN STATE, NIGERIA.**

**QUESTIONNAIRE ON EXCRETA MANAGEMENT PRACTICES AND DIARRHOEAL ILLNESSES AMONG UNDER-5 CHILDREN IN ILE IFE, OSUN STATE, SOUTHWEST NIGERIA.**

**Introduction:**

This questionnaire is for research on excreta management of under five children by their caregivers. The aim of the study is to undertand your knowledge and guidance of under-five children in faeces management. This study is for leaning and the information provided will be confidential. You have been selected among many eligible caregivers and we sincerely thank you for your co-operation and true responses to the questions in all the sections.

**SECTION A: Socio-demographic and -economic characteristics of respondents**

1. Age in completed years (as at last birthday):___________________
2. Sex: (a) Male [ ] (b) Female [ ]
3. Religion: (a) Christianity [ ] (b) Islam [ ] (c) Traditional [ ] (d) Others ( specify)_______
4. Ethnicity: (a) Yoruba [ ] (b) Igbo [ ] (c) Hausa [ ] (d) Others (specify)__________
5. Your marital status: (a) Single [ ] (b) Married [ ] (c) Divorced [ ] (d) Separated [ ] (e) Widow/widower [ ]
6. Type of marriage (a) Polygamous [ ] (b ). Monogamous [ ] (c) Single parenthood [ ]
7. Number of household members: _______b. Number of children ever born:___________
8. How many Under 5 children do you have?:____ b. Their ages (yrs):____;____;____;____
9. Your relationship to the under-5 child (ren). a. Mother [ ]. b grand mother [ ]. c. Paid carer [ ]. d. House help [ ]. e. Others (specify)____________________
10. Number of people that slept in your household last night:_______b. No of bed rooms:______
11. Your highest educational qualification: (a) No formal education (b) Some primary [ ] (c) Completed primary [ ] (d) Some secondary [ ] (e) Completed secondary [ ] (f) Tertiary [ ] (g) Vocational [ ] (h) Quranic Education [ ]
12. Your husband’s educational qualification (pick option from question 10):_________
13. Your main occupation: (a) Business/commerce [ ] (b) Civil servant [ ] (c) Semi skilled (hardresser/labourer/ Petty trading) [ ] (d) Daily paid artisan/mason [ ] (e) Farming [ ] (f) Unemployed/student [ ] (g) Full-time housewife [ ] (h) Monthly Private sector paid employment [ ] (i)Pensioner/retired [ ] (j) Others(please specify)___________________
14. Family monthly income/profit (Naira): a. Respondent:_______b. Husband:______

| 15. Household and wealth indices of respondents *(Yes =1, No=0)* | Yes | No |
| --- | --- | --- |
| i. Household owns the house |  |  |
| ii. House walls built with blocks |  |  |
| iii. House floor well cemented and/or tiled |  |  |
| iv. Household has working/functional fridge/freezer |  |  |
| v. Household has working/functional TV/video |  |  |
| vi.Household has paid satellite TV (e.g Dstv/Go-TV/Star-time, etc) |  |  |
| vii. Household owns car/motor cycle |  |  |
| viii. Household own generator/desktop or laptop computer |  |  |
| ix. Household have separate room for kitchen? |  |  |
| x. Household share the same bathroom/toilet with____(how many) households |  |  |
| xi. Household uses gas/electric cooker/kerosine for cooking |  |  |

**SECTION B: Knowledge of mothers on sanitation and the management of faeces passed by under five children**

2.1. Which of the following statements is correct about how a child’s faeces should be handled? (a). left on the soil in the household premises for domestic animals to eat [ ] ( b). Packed and disposed in nearby open dump [ ] c. Packed and disposed in toilet [ ]. d. Packed and disposed with household solid waste [ ].

2.2. The faeces of under-5 children should be used in farms immediately as manure for plants growth. A. Yes [ ] b. No. [ ] c. I do not know [ ].

2.3. The following are ways of training a child to safely defecate, except. A. on land when pressed [ ]. B. In potty [ ]. C. In childs toilet [ ]. D. In sanitary napkins [ ].

2.4. Which among the following is the correct process in hand washing? (a). Water soap washing rinsing personal hand towels/air dry [ ]. (b). Water washing rinsing air dry [ ]. (c). Water washing personal hand towels [ ]. (d) Soap water washing rinsing air dry [ ].

2.5. Hand washing should be performed at each of the following critical times, except a. After I used the toilet [ ] b. After cleaning up the under-5 child after defecation [ ] c. Before i feed my child [ ]. d. After feeding the under-5 child [ ] e. All of the above

2.6. Faeces ofunder-5 children should be disposed at which of the following times? a. immediately after defecation b. after about 5 minutes c. at the caregiver’s convenience d. All of the above.

2.7. Child’s potty should be kept A. close to the kitchen [ ] B. in the corridor/passage [ ] C. in the toilet [ ] d. Around the toilet/bathroom [ ]

2.8. Which of the following diseases can under-5 children have when their faeces are not properly managed? A. Malaria [ ] B. Tuberculosis [ ] C. Cholera [ ]. D. measles [ ]

2.9. A child’s potty should be washed with water only after use a.Yes [ ]. b No [ ] c Not sure [ ]

2.10. The faeces of Under-5 children is harmless A. Yes [ ] B. No [ ] C. I dont know [ ]

**SECTION C: Attitude of respondents on handling and management of excreta passd by under-five chilren.**

**Instruction**: Please tick your most appropriate response for each of the questions in this section.

| Questions statements | Strongly disagree | Disagree | Neither agree nor disagree | Agree | Strongly agree |
| --- | --- | --- | --- | --- | --- |
| 1. Having an improved toilet, that is safe to use is not mandatory since my child can defecate in sanitary napkins and on soil? |  |  |  |  |  |
| 1. Safe disposal of under-5 children faeces in toilet, cleaning & washing their bottom and hands with soap & water afterwards is tiring? |  |  |  |  |  |
| 1. Child faeces is not harmful at all, when compared to adult’s faeces. |  |  |  |  |  |
| 1. There is no need for hand washing with soap and water after handling child’s faeces. |  |  |  |  |  |
| 1. It is advisable to keep the under-5 child’s potty close to your source of water supply to wash thoroughly. |  |  |  |  |  |
| 1. It’s not necessary to wash child’s hands with soap and water after defecation since the child’s bottom/anus has been washed. |  |  |  |  |  |
| 1. Child’s faeces should be disposed immediately after defecation |  |  |  |  |  |
| 1. Children should be potty trained at infancy |  |  |  |  |  |
| 1. I (you) can ensure safe disposal of children faeces even when I do not have a toilet in the house |  |  |  |  |  |
| 3.10. Buying a potty is a waste of money when the #  child can defecate around in the household in our  compound? |  |  |  |  |  |
| 3.11. Disposing child faeces on open dunps can lead to  diarrhoeal diseases? |  |  |  |  |  |

**SECTION D: Sanitation and child excreta management practices**

1. Your main source of domestic water is (a). Pipe water into dwelling. (b). Pipe water into premises. (c). Public tap/standpipe. (d). Motorised borehole/Solar-powered borehole/ Hand-pump borehole. (e). Protected dug well/protected spring. (f). Rainwater harvesting. (g). Unprotected/open dug well/Undeveloped spring. (h). Bottled/sachet water. (i). Cart supply with kegs/drums/tanker supply. (j). Surface water (river/dam/lake/ pond/stream).
2. How long does it take to go to the main water source, fetch water and return home? a. within the premises b. Delivered to home c. Offsite __________minutes. d. I dont know
3. The type of toilet facility in your household is (a). Water closet flushed into sewer/ septic tank/pit [ ]. (b). Water closet flushed elsewhere. (c). Pour flush system into sewer/ septic tank/pit. (d). Pour flush system elsewhere. (e). VIP latrine. (f). Improved pit latrine with concrete slab. (g). Pit latrine with wooden slab. (h). Open pit/latrine. (i). Bucket latrine/Hanging toilet. j. No facility/bush/field/in water [ ]
4. Location of the toilet? a. Within the flat b. Within the house. c. Ouside the house/compound d. None
5. Distance of latrine from household: a. Within the flat _b._within the house. c. outside the house:___meters
6. Who routinely care for the under 5 chil(ren) on defecation ? a. Mother [ ]. b. Grandmother. c. Adolescent girl d. House help e. Anybody in the house. f. Others, specify______________
7. What is the usual place for the child to pass stool ***during the day***? (a) diaper/clothes (b) nappy (c). sanitary napkin (d). Paper/nylon (e). On soil in the compound (f) in potty (f) in his/er toilet seat (g) anywhere (h) others (specify)____________
8. What is the usual option for the child to pass stool ***at night***? (a) in nappy (b) in sanitary napkin (c). on paper /nylon (d). In potty (f) in his/er toilet seat (g) in childrens toilet (h) others, specify__
9. What determines the child defecation strategy in use by you? a. Convenience b health c. cost. d.best affordable
10. How do you transport the child’s faeces? a potty b. paper c. nylon d. shovel/hoe e. does not apply
11. How do you dispose the child’s faeces? (a) left in the open in the compound (b) latrine/ toilet (c) packed in the household solid waste storage receptacle (d) disposed in nearby bush/open dump (e) open drainage ditch (f) domestic animals feeds on it
12. How do you clean the child’s bottom/anus after defecation? (a) wipe with bare hands, (b) wipe with a clean section of the soiled nappy/sanitary napkin, (c) use paper, (d) use cloth. (e). rinse with water (f) use tissue paper/paper. (g) Child bottom not usually cleaned after defecation (h) others (specify)_____
13. How is the child’s anal cleansing material routinely disposed after use? (a) cleaning water disposed/soaked in the envirnment (b) disposed in open dumps (c) disposed with domestic solid waste (d) disposed in toilet (e) No specific disposal method (f) others, specify______________________
14. How do you clean your hands after contact with child’s faeces? (a) Do not clean/wash hands after caring for the child (b) wash hands with water only (c) wash hands with water and soap (d) clean hands with rags only.
15. In your household, do you have a dedicated tool/material to clean up your child’s faeces around your house after defecation? a.Yes [ ] b.No [ ]
16. If yes to question 10 what tool do you use?________________________________
17. If yes to question 10, do you wash the tool after use . Yes [ ] b.No [ ]
18. If yes to question 10, with what do you wash the tool? a. water only [ ] b. soap and water [ ] c.

Others, specify________

**Administer as apprpriate to the under-five child (ren): questions 4.17 to 4.18.**

| Question | **Not crawling** | **Crawing** | **Walking with assistance** | **Walking unaided** |
| --- | --- | --- | --- | --- |
| **4.1.7 t**he last time that the child passed stool, where did S/he did it? (a) latrine/toilet (b) potty (c) nappy (d) paper (e) nylon (f) on ground inside the household (g) on soil within the compound/premises (h) in the nearby open dump (i) Others,specify:________________ |  |  |  |  |
| **4.1.8.** The last time the child passed stool, how was it disposed? (a) put/rinsed into latrine/ toilet (b) stored/ disposed with solid waste (c) burriedin the premises (d) left in the open (e) fed to domestic animals (f) diluted with water in the premises (g) others, specify______________ |  |  |  |  |

4.19. What is the ideal place you would like your child to defecate?___________________________

4.20. Why is this (the option above) the ideal place you would like your child to defecate?_________________

**SECTIN E: Common faeco-oral diseases among under-five children**

Instruction: Tick either ***yes or no if your under-five year old (index) child had) any of the following diseases wihin the last 2-weeks***

| Possible diseases | Yes | No | Mentioned signs & symptoms | Frequency |
| --- | --- | --- | --- | --- |
| 1. Diarrhoea |  |  |  |  |
| 2. Dysentry |  |  |  |  |
| 3. Typhoid |  |  |  |  |
| 4. Cholera |  |  |  |  |
| 5. Intestinal worm infestation |  |  |  |  |

*NB: Frequency: Number. of times within 2-week period?*

**SECTION F : caregivers’ Information and communication preferences**

6.1.Have you ever received information/message on safe excreta disposal forhouseholds? Yes [ ] b.No [ ]

6.2.Have you ever received information/message on safe excreta disposal for under-five children? Yes [ ] b.No [ ]

| **Message(s) sources** | **Focus of the message** | | | |
| --- | --- | --- | --- | --- |
|  | **Household** | | **Child** | |
| 6.3. Identify the various sources of information (Tick all that apply)? | Yes | No | Yes | No |
| i. Town Announcers |  |  |  |  |
| ii. SMS on hand-held, mobile phone |  |  |  |  |
| iii. Posters in healthcare facility |  |  |  |  |
| iv. Hand bills |  |  |  |  |
| v. Billboard |  |  |  |  |
| vi. Friends |  |  |  |  |
| vii. Health talks at healthcare facility |  |  |  |  |
| viii. Radio talk show/annnouncement |  |  |  |  |
| ix. Television talk show/announcement |  |  |  |  |
| x. Newspaers (Yoruba or English) |  |  |  |  |
| xi. Others (specify |  |  |  |  |

6.4. Do you think you need more knowledge about safe management of faeces for under 5 children? A.Yes. [ ]. B.. No [ ] c. I don’t know [ ]

6.5. List two areas where you required additional knowledge on the management of child faeces.

1. ___________________________________________________________________

2. ___________________________________________________________________

**SECTION G: Observational Checklist (Section 1: child defecation)**

7.1 Child defecated while in the household. Yes [ ] b.No [ ]. If No, go to question 7.16.

7.2. How mobile is the child (a. immobile, 2. crawling, 3. walkingaided. 4 walkingunaided

7.3. Child defecation site, if any. (a) household floor/premises (b). Potty (c). Children’s/household toilet (d). Open dump (e) Others, specify__________

7.4. Transport of faeces. Did anyone pick up/remove the stool after defecation? Yes [ ] b.No [ ].

7.5. How was the faeces transported? (a). Not transported but covered with soil (b) by hand (c) cutlass (d) hoe (e) paper/nylon (f) potty (g) others, specify________________________

7.6. Where was it transported to? (a) toilet (b) open dump/bush (c) open drain (d) solid waste storage reservoir (e). Others specify________

7.7. Was(were) the transportation/diposal tool(s) cleaned after use? Yes [ ] b.No [ ]. Not observed [ ].

7.8 With what were the tools cleaned after use? a.Not cleaned. b.Soil c.Water d.Water & soap

7.9. was the child anus/bottom cleaned after defecation? Yes [ ] b.No [ ].

7.10. How was the child’s bottom cleaned? a.With water alone b. with soap & water. c.wiped with cloth. d.wiped with clean part of soiled napkin

7.11. Were the child’s hands cleaned ? Yes [ ] b.No [ ]

7.12. With what were the child hands cleaned? a.with soap and water. b. wiped with cloth. c. wiped with clean part of soiled napkin

7.13. After finally disposing the child faeces, does the caregiver clean hands? Yes [ ] b.No [ ].

7.14. if yes, with what did the carer clean hands? a.with soap and water. b.wiped with cloth. c.wiped with clean part of soiled napkin, d. Others, specify___________________________

7.15. How was the material used to clean the child disposed? a. disposed in the environment. b. washed for reuse. c. left in the open in the premises. d. Others, specify.________________

7.1.6 The latrine/toilet have (1) superstructure: Yes [ ] b.No [ ]; (2) Roofed: Yes [ ] b.No [ ]; (3) door: Yes [ ] b.No [ ]; (4) inside lock: Yes [ ] b.No [ ]; (5) odour: Yes [ ] b.No [ ]; (6) house flies: Yes [ ] b.No [ ]; (7) faecal drops: Yes [ ] b.No [ ]; (8) well maintained: Yes [ ] b.No [ ]

**Section 2: Presence of excreta in the surounding**

8.1 Child faeces present in the respondent premises/compound Yes [ ] b.No [ ]

8.2. Where was the faeces present? a. around the main water source/reservior. b. on soil c. aound the kitchen. d. aound the toilet, e. around the shop f. Others, specify_____________

8.3. Flies houvering around the faeces? Yes [ ] b.No [ ]

8.4. open adult food source around? Yes [ ] b.No [ ]

8.5. open child food around? Yes [ ] b.No [ ]
